# Convolutional neural networks on risk stratification of patients with suspected coronary artery disease undergoing coronary computed tomography angiography

**DOI:** 10.1007/s10554-023-02824-y

**Published:** 2023-04-03

**Authors:** Rafael Adolf, Nejva Nano, Alessa Chami, Claudio E. von Schacky, Albrecht Will, Eva Hendrich, Stefan A. Martinoff, Martin Hadamitzky

**Affiliations:** 1 Department of Radiology and Nuclear Medicine, German Heart Center Munich, Technical University of Munich, Lazarettstrasse 36, 80636 Munich, Germany; 2 Department of Diagnostic and Interventional Radiology, Klinikum München Neuperlach, Munich, Germany; 3 Department of Diagnostic and Interventional Radiology, Klinikum Rechts der Isar of Munich Technical University, Munich, Germany

**Keywords:** Convolutional neural networks, Coronary computed tomography angiography, Prognosis, Coronary artery disease

## Abstract

To assess the prognostic value of convolutional neural networks (CNN) on coronary computed tomography angiography (CCTA) in comparison to conventional computed tomography (CT) reporting and clinical risk scores. 5468 patients who underwent CCTA with suspected coronary artery disease (CAD) were included. Primary endpoint was defined as a composite of all-cause death, myocardial infarction, unstable angina or late revascularization (> 90 days after CCTA). Early revascularization was additionally included as a training endpoint for the CNN algorithm. Cardiovascular risk stratification was based on Morise score and the extent of CAD (eoCAD) as assessed on CCTA. Semiautomatic post-processing was performed for vessel delineation and annotation of calcified and non-calcified plaque areas. Using a two-step training of a DenseNet-121 CNN the entire network was trained with the training endpoint, followed by training the feature layer with the primary endpoint. During a median follow-up of 7.2 years, the primary endpoint occurred in 334 patients. CNN showed an AUC of 0.631 ± 0.015 for prediction of the combined primary endpoint, while combining it with conventional CT and clinical risk scores showed an improvement of AUC from 0.646 ± 0.014 (based on eoCAD only) to 0.680 ± 0.015 (p < 0.0001) and from 0.619 ± 0.0149 (based on Morise Score only) to 0.6812 ± 0.0145 (p < 0.0001), respectively. In a stepwise model including all prediction methods, it was found an AUC of 0.680 ± 0.0148. CNN analysis showed to improve conventional CCTA-derived and clinical risk stratification when evaluating CCTA of patients with suspected CAD.

## Introduction

Coronary computed tomography angiography (CCTA) has been widely incorporated into the clinical setting as a first line strategy in ruling out obstructive coronary artery disease (CAD) in patients with low to intermediate risk [1].

Beyond the identification and grading of coronary artery stenosis, it allows the characterization of atherosclerotic plaque features that have prognostic implications such as low-attenuation plaque, spotty calcification, napkin-ring sign and remodeling [2, 3].

Semi-automatic and semi-quantitative evaluations of high-risk plaques have been extensively developed for several years. However, CCTA-based risk assessments are not yet taken regularly into account in the clinical decision-making process, mainly because it demands quite a time expenditure of highly trained professionals for a still limited additional benefit compared to other risk prediction models.

With the rise of automatic machine learning (ML) algorithms including deep learning (DL) there is an expectation of improvement in diagnosis and prognostication for patients with cardiovascular diseases [6]. The identification of plaque features using ML tools has been shown already to outperform conventional quantitative and qualitative CCTA analysis [4–6].

A type of deep-learning algorithm, the so called convolutional neural networks (CNN), has been developed to process imaging data exhibiting natural spatial invariances [7]. Using a training sample of images, it is able to learn features from images and execute tasks such as labeling an image to a group or class, detecting an object or generating a new image, so that CNN is considered nowadays the state of the art in image analysis [8, 9].

With the increasing importance of diagnostic imaging and the rapid expansion of medical recorded data, CNN may be helpful in evaluating computed tomography datasets more effectively and has the potential to even recognize imaging patterns that the human eye can not see in traditional grayscale computed tomography (CT) scans.

Therefore, the aim of our study was to evaluate the long-term prediction of major cardiovascular events using CNN on CCTA-images of patients with suspected CAD in comparison with clinical and conventional CCTA-based risk scores.

## Materials and methods

### Study population

In this study, we enrolled 5468 consecutive patients who underwent CCTA for suspected coronary artery disease (CAD) at the German Heart Center in Munich, Germany from October 2004 to January 2018.

Patients with acute coronary syndrome, presence of a life-threatening situation, a lack of stable sinus rhythm during the examination, prior stent implantation or coronary bypass surgery were excluded from analyses. Before examination, a structured interview was performed, including patient age, height and weight, as well as history of cardiac disease, present concerns and current medication.

Laboratory results and cardiac risk factors were assessed. The pretest probability of CAD was calculated using the Morise score [10], which includes age, gender, risk factors and symptoms to predict the probability of obstructive CAD. According to the number of coronary arteries with obstructive CAD (defined as ≥ 50% stenosis) the extent of coronary artery disease was classified as 0-, 1-, 2- or 3-vessel disease.

Follow-up information was gathered either through clinical visits, questionnaires sent by mail or phone contact. Of the 7770 patients initially enrolled in the study, 5605 could be reached for clinical follow-up. 25 patients had to be excluded due to absent individual cardiovascular risk factor values and further 137 individuals had missing or non-diagnostic images. Primary combined endpoint of the study consisted of major adverse cardiac events (MACE) defined as composite of all-cause death, myocardial infarction, unstable angina, or late revascularization (> 90 days after CCTA).

Training endpoint included additionally patients undergoing coronary revascularization within 90 days after CCTA and was used together with the primary endpoint in the two-step training of the full network.

### Image acquisition

**Fig. 1:**
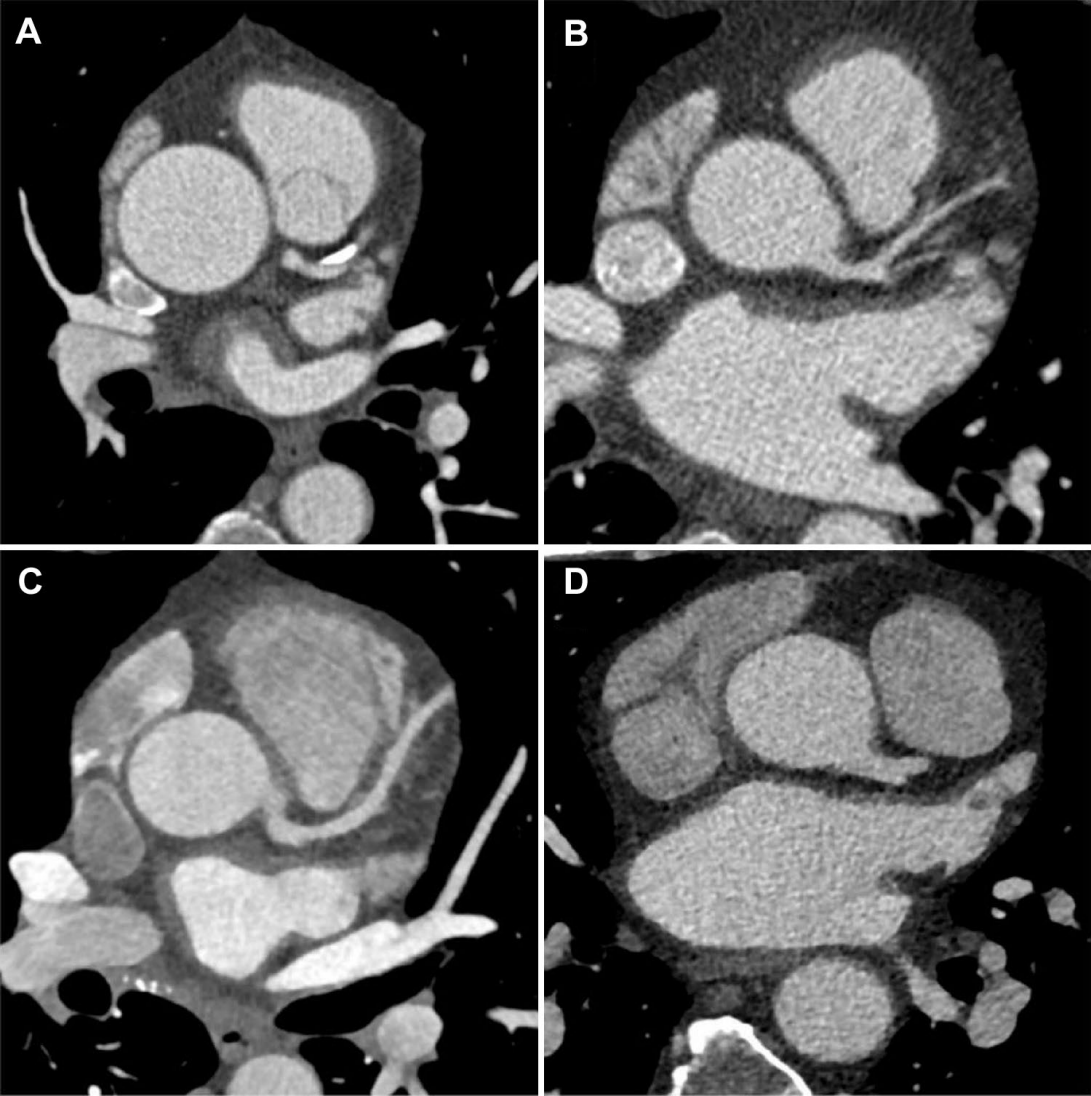
CCTA images of 4 different CT generations used for acquisition throughout the study period. Image **A**: 64-slice single source CT; Image **B**: 64-slice dual source CT; Image **C**: 128-slice dual source CT; Image **D**: 128-slice dual source CT

Throughout the study period 4 different CT scan generations were used for image acquisition (Fig. 1).

A 64-slice single source CT system from October 2004 to September 2006, a 64-slice dual source CT system from October 2006 to March 2009, a 128-slice dual source CT system from April 2009 to May 2014, and a 192-slice dual source CT system from June 2014 to January 2018 (all Siemens Medical Solutions, Erlangen, Germany).

According to the patient’s heart rate and absence of contraindications intravenous beta-blocker medication was administered targeting a heart rate less than 60 beats/min. Sublingual nitrates were applied if systolic blood pressure was higher than 100 mmHg.

The coronary prospective ECG-synchronized CTA was triggered into the diastolic phase (70% of RR-interval). Tube voltage was selected by the technician and/or physician between 70 and 120 kVp, tube current was adapted automatically based on body size (CARE Dose). Contrast circulation time was determined using a testbolus with 10 ml contrast media (Imeron 350, Bracco Imaging GmbH, Konstanz, Germany), followed by a 50 ml 0.9% saline chaser. The coronary CT angiogram was performed with a 50 ml contrast bolus at 5.0 ml/s, followed by 30 ml 0.9% saline chaser.

Axial thin slice images were reconstructed with 0.6 mm slice width and increment of 0.4.

### Plaque assessment from CCTA

Coronary artery luminal stenosis was evaluated and interpreted by at least two experienced radiologists and graded as none (0%), minimal (1–24%), mild (25–49%), moderate (50–69%), and severe (≥ 70%). Coronary artery plaques were characterized as non-calcified only, predominantly non-calcified, predominantly calcified or calcified only.

### Image annotation and preprocessing

The 3D dataset was analyzed using a commercially available software (Syngo.via, Siemens Healthineers, Erlangen, Germany) and the coronary artery tree was segmented automatically with manual correction of inconsistencies. This yielded centerlines and a mask denoting vessel lumen and vessel wall including plaques for all detectable vessel branches. The vessel regions containing non-calcified and partially calcified plaques were marked manually, calcified plaques were annotated automatically using a threshold algorithm: along the centerline of each segment mean and maximum contrast intensity was calculated. Calcification was marked, if pixel intensity was more than 150HU above maximum vessel contrast. To correct for outliers, maximum contrast was limited to 120% of mean contrast.

**Fig. 2:**
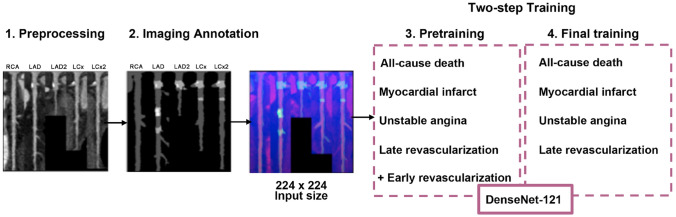
Model architecture overview. Initially (step 1), coronary artery segments were reformatted in multi angle stretched curved planar reconstructions (SCPR). Next (step2), images were integrated with the annotation mask of non-calcified, partially calcified and calcified, as well as distance from vessel ostium. 224 × 224 matrix was used as input for an ImageNet pretrained DenseNet-121 with a two-step training: first, the full network was trained using the training endpoint (step 3), which included early revascularizations; then, the feature layer was further trained using only the primary endpoint (step 4)

Coronary arteries were reformatted into 2D multi angle images as stretched curved planar reconstructions (SCPR). Up to five reformations (1 for RCA, 2 for LAD, and 2 for LCx territory) were then integrated in one image with a 224 × 224 matrix holding each pixel data, annotation mask, and distance from vessel ostium in one color channel (Fig. 2), for each patient 36 reconstructions of different angles around the centerline were calculated.

### Model architecture and model training

An ImageNet DenseNet-121 a binary classification layer was used.

The whole dataset was split randomly into five groups stratified by scanner generation, both endpoints, gender and age (dichotomized by median).

Hyperparameter optimization was done on a 4:1 training-validation-test split. Hyperparameters are listed in Table 1. The parameters selected for the main training are marked bold. In the optimized configuration 72 images per patient were used resulting on average in 38,477 training images and 9629 validation images.

**Table 1:** ImageNet DenseNet-121

Hyperparameter	Values tested
DenseNet Model type	**121**, 161, 169, 201
Usage of pretrained Model	**Yes**, no
Optimizer	Adam
Batch size	4, 8, **16**
Epochs	12, **24**, 36, 64
Image augmentation, horizontal flip	**Yes**, no
Image augmentation, number of stretched CPRs	9, 18, **36**
Percentage of positive endpoints	25, 33, **50**
Sets with different negative endpoints	1, 2, 4, **6**, 12
Learning rate, initial value	0, 001.0, 01, **0, 1**, 0, 2, 0.5
Learning rate, gamma	0.02, 0.05, **0.1**, 0.2
Learning rate, step	4, **8**, 12

The final results were acquired using five time cross validation with one group serving as validation group and the other as training group.

Densenet models are pretrained on nonmedical image. These models failed to converge on the clinical endpoint. We therefore chose a two-step approach. First the network was trained using the training endpoint, which included early revascularization; then it was further optimized using the primary endpoint.

Model training was performed in Python 3.8.5 (open-source; Python Software Foundation, Wilmington, Del) by using pytorch 1.7.1 and scikit-learn 0.23.2 libraries on a GPU-workstation with a 8-core Intel-Core i7 9700 K-CPU at 3.6 GHz (Intel, Santa Clara, Calif), 64 GB DDR4-SDRAM and 4 GeForce RTX 2080ti 11 GB graphical processing units (Nvidia, Santa Clara, California) running Linux system (Ubuntu 20.04; Canonical, London, England) with CUDA 11.3 (Nvidia, Santa Clara, CA).

### Statistical analysis

The prediction of the fully trained model, normalized by the softmax function was used as a variable for further statistical tests. Outcome prediction and incremental value compared to the extent of CAD was done by receiver operating statistics. All statistical tests were performed two-sided and a significance level of 5% was used. The statistical package R version 2.10.1 including the package rms was used for statistical analysis.

## Results

A total of 5468 patients were included, with a mean age of 61.1 ± 11.2 years, and 66.5% were male. In total, 334 primary endpoint events (168 deaths, 27 non-fatal myocardial infarction, 1 unstable angina and 154 late revascularization) occurred during a median follow-up duration of 7.2 years. Additionally early revascularizations occurred in 405 (7.4%) patients. Table 2 shows the characteristics of the study participants.

**Table 2:** Patient characteristics (*n* = 5468)

Demographics	
Age (years)	61.1 ± 11.2
Male sex, n (%)	3637 (66.5)
Body-mass-index (kg/m^2^)	24.8 ± 42.1
CAD risk factor	
Diabetes, n (%)	419 (7.6)
Smoking, n (%)	1757 (32.1)
Hypertension, n (%)	2983 (54.6)
Hypercholesterolemia, n (%)	2992 (54.8)
CAD family history, n (%)	1885 (34.5)
CAD risk scores	
Morise risk score	11.1 ± 2.74
No CAD (%)	1108 (20.3)
No-obstructive CAD (%)	2994 (54.7)
Obstructive CAD (%)	1366 (25)
1	958 (70.1)
2	307 (22.5)
3	101 (7.4)
CAD-RADS	
0	1108 (20.2)
1	1111 (20.3)
2	1883 (34.4)
3	1056 (19.3)
4a	261 (4.8)
4b	22 (0.4)
5	27 (0.5)

Data presented as mean ± standard deviation or absolute number (percentage)

Of the 5,468 patients, 419 (7.6%) showed diabetes, 1757 (32.1%) were currently or had a history of smoking and 1885 (34.5%) had a positive family history of cardiovascular disease (CAD). Hypercholesterolemia was found in 2992 (54.8%) patients and hypertension in 2983 (54.6%) patients. The study population showed an average Morise risk score of 11.1 ± 2.74. No CAD was observed in 1108 patients (20.3%), 2994 patients (54.7%) were diagnosed with non-obstructive CAD, and 1366 patients (25%) showed obstructive CAD. Baseline differences in terms of cardiovascular risk factors between groups with or without the occurrence of primary and training endpoint are shown in Table 3.

**Table 3:** Baseline differences between groups with and without the occurrence of primary endpoint

	Negative n = 5,134	Positive n = 334	p value
Age (years)	60.8 ± 11.1	66.8 ± 10.8	< 0.0001
Male sex, n (%)	3397 (66.2)	240 (71.9)	0.036
Hypertension	2725 (53.1)	220 (65.9)	< 0.0001
Smoking, n (%)	1641 (32)	116 (34.7)	0.3
Diabetes, n (%)	378 (7.36)	41 (12.3)	0.002
Hypercholesterolemia, n (%)	2802 (54.6)	181 (54.2)	0.91
CAD family history, n (%)	1783 (34.7)	102 (30.5)	0.12
Total cholesterol mg/dl	206 ± 50.6	201 ± 56	0.086
LDL	128 ± 38.7	126 ± 41.3	0.49
HDL	59.7 ± 24.2	55.1 ± 17	< 0.0001
Triglicerides	142 ± 106	146 ± 90.8	0.48
Morise risk score	11.1 ± 2.74	12.2 ± 2.58	< 0.0001
Low	911 (17.8)	20 (6.01)	
Intermediate	3984 (78)	274 (82.3)	
High	215 (4.21)	39 (11.7)	
eoCAD	1.02 ± 0.668	1.4 ± 0.625	p < 0.0001
No CAD (%)	1083 (21.1)	25 (7.49)	
Non-obstructive (%)	2843 (55.4)	151 (45.2)	
Obstructive (%)	1208 (23.5)	158 (47.3)	

Data presented as mean ± standard deviation or absolute number (percentage)

*CAD* coronary artery disease, *eoCAD* the number of obstructive vessels on CTA

The primary and secondary endpoints are shown in Table 4.

**Table 4:** Primary and secondary endpoints

Events	
All-cause death^a^	169 (3.09)
Cardiac death	97 (1.77)
Non-cardiac death	72 (1.32)
Myocardial infarction^a^	27 (0.494)
Unstable angina^a^	1 (0.0183)
Coronary artery bypass graft surgery	32 (0.585)
Early coronary artery bypass graft surgery	17 (0.311)
Late coronary artery bypass graft surgery	15 (0.274)
Percutaneous coronary intervention	572 (10.5)
Early PCI	428 (7.83)
Late PCI	144 (2.63)
Revascularization	599 (11)
Early revascularization	445 (8.14)
Late revascularization^a^	154 (2.82)

Data presented as absolute number (percentage)

*PCI* Percutaneous coronary intervention

^a^Combined primary endpoint

CNN based risk prediction for primary endpoints had an area under the curve (AUC) of 0.631 ± 0.015. Regarding the training endpoint it was observed an AUC of 0.720 ± 0.010 with the CNN algorithm. When combining CNN analysis with CT-based parameters, we found an improvement of AUC to predict primary endpoints from 0.646 ± 0.014 (based on eoCAD only) to 0.680 ± 0.015 using CNN in addition to eoCAD (p < 0.0001). Clinical risk assessment using the Morise score demonstrated an AUC of 0.619 ± 0.0149 for predicting the combined primary endpoint, while combining it with CNN showed an increased AUC of 0.6812 ± 0.0145 (p < 0.0001).

**Fig. 3:**
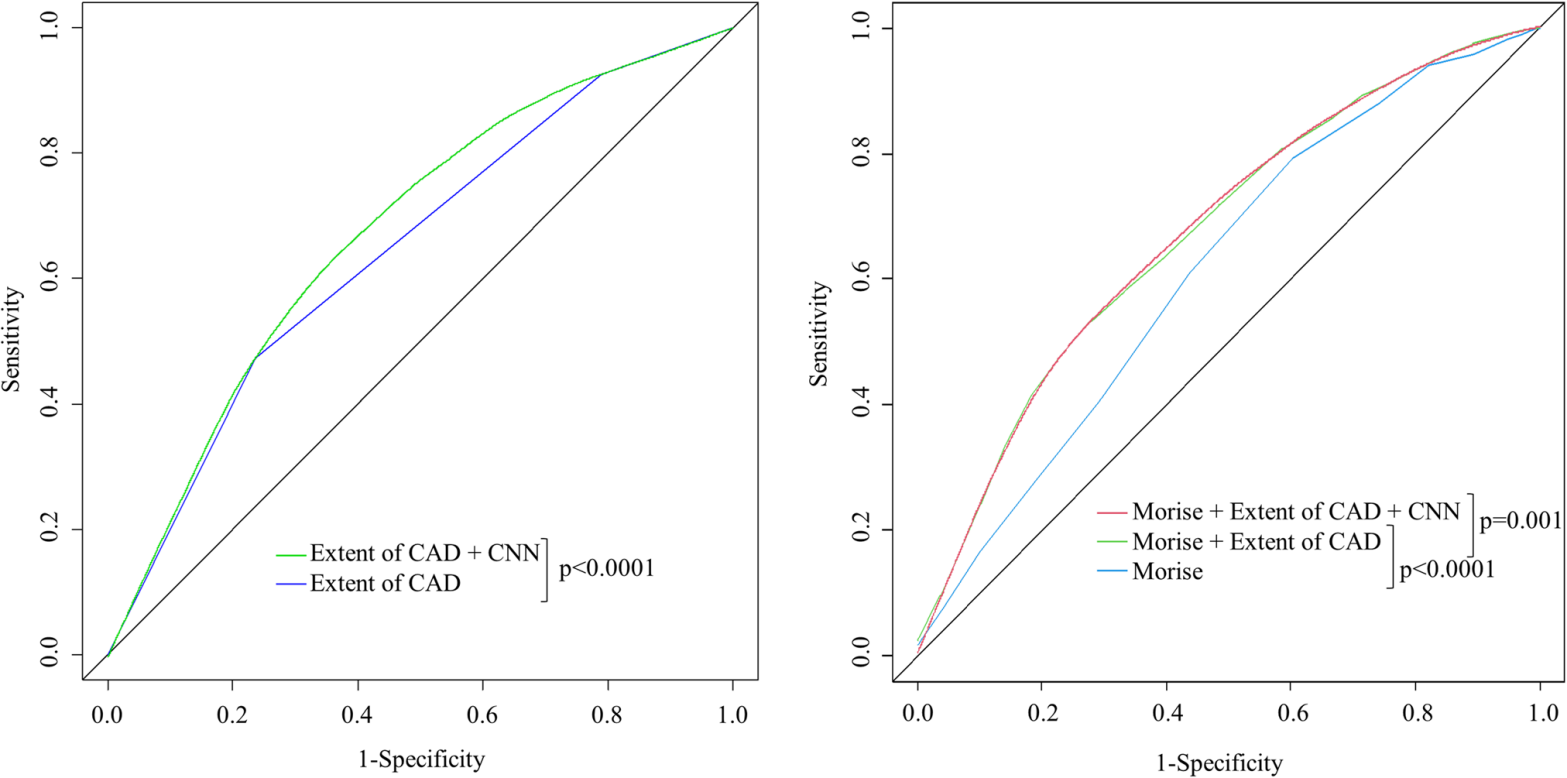
ROC-Curves for Prediction of Major Cardiovascular Events

In a stepwise model combining all prediction methods, it was found an AUC from 0.619 ± 0.0149 for Morise score alone, increasing to 0.676 ± 0.015 after adding eoCAD (p < 0.0001) and, eventually, to 0.680 ± 0.0148 by means of Morise score, eoCAD and CNN combined together (p = 0.0001) (Fig. 3).

## Discussion

Our study shows an improved risk prediction for MACE in patients undergoing CCTA combining CNN with conventional CT parameters and clinical risk factors. These results highlight the potential of integrating machine learning (ML)-based image analysis into the evaluation of coronary plaque features in order to improve prognostication of patients with suspected CAD.

To our knowledge, this is the first approach to use a CNN algorithm directly on risk assessment of patients with suspected CAD. Up to now, ML-based models were used to optimize prediction based on known plaque features and CNNs were used to automate the detection of these features. Our CNN model was fed with only a scarce amount of information about the coronary plaque characteristics and was able to enhance the prognostication of MACE evaluating not 3D CT data, but merely 2D integrated images of the coronary arteries.

Previously, ML-based models in cardiac CT imaging were either used to optimize prediction of cardiovascular outcome or to simply automate and enhance morphologic plaque characterization. CCTA-based qualitative and quantitative plaque features were used by Al’Aref et al. to create a ML model to predict culprit lesions among acute coronary syndrome (ACS) patients and showed a significantly higher AUC when compared to models based on high-risk plaque features, diameter stenosis and lesion-level plaque analysis [11]. This model also demonstrated a specificity of 89% for predicting non-culprit lesions in patients who underwent CCTA without presenting acute coronary syndrome. Motwani et al. [12] analyzed clinical and CCTA-based risk scores for the prediction of 5-years all-cause mortality and found an improved AUC using ML when compared to Framingham risk score (FRS) or CCTA data alone.

Our group performed a ML-based time-to-event analysis in a similar cohort of patients with suspected CAD [13], which showed a superior performance for the long-term prediction of MACE than the use of clinical and CCTA derived variables or scores, independently.

In a multicentric study, Lin et al. [14] developed and externally validated a deep learning based algorithm to measure total plaque volume and minimal luminal area that correlated closely with expert reader measurements and intravascular ultrasound. However, an association between an increased risk of myocardial infarction and deep learning-based total plaque volume could only be shown after adjustment for clinical risk scores and the presence of obstructive stenosis.

Using a multi-task recurrent convolutional neural network (RCNN) Zreik et al. [15] demonstrated the feasibility of an algorithm for an automatic detection and characterization of coronary plaques and stenosis. This method showed a high accuracy in detecting and determining the significance of coronary stenosis but only a moderate reliability in classifying coronary plaques, as the differentiation of the mixed plaque from the calcified and non-calcified plaques remains a major challenge.

The main focus in applying neuronal networks to X-ray coronary angiography (CAG) is automated stenosis detection and characterization. In CT angiography, there are several commercially available systems, but their algorithms are not known in detail. In invasive coronary angiography Stralen et al. compared three CNNs for stenosis detection in the right coronary artery in 9278 invasive angiography and identified EfficientDet D3 as the best performing model [16]. Cong et al. compared different CNN architectures for classifying stenosis as < 25% or > 25% based on QCA-data from 230 invasive angiographies and identified Inception-v3 as best performing model [17].

The aim of this study was not to automatically detect single image parameters but to use the clinical outcome as ground truth and the main challenge was to adapt one of the many available CNNs to this new endpoint and the additional variance. DenseNet family was chosen because of the relatively large size of the input matrix, its usage in other studies in the field and the good performance in classification of non-medical images [18, 19].

Reliable risk assessment based on coronary plaque features is challenging as quantitative and qualitative analysis softwares are often time-consuming with more than 40 different plaque characteristics to be considered. Even after years of development, semi-automatic plaque evaluations still show restricted inter- and intraobserver agreement, especially in patients with higher coronary disease burden when evaluating calcified and low-attenuation plaques [4, 20–22]. Additionally, plaque analysis is not performed in a strictly standardized fashion among different research centers, since acquisition protocols, CT scans, software algorithms and levels of experience of CT readers may differ between medical care centers.

The good performance of the new ML algorithms emphasizes the complex nature of plaque analysis where different parameters carry only a fraction of the prognostic information. Assuming that relevant prognostic information lies in the coexistence of different parameters and part of this is still unknown, we tried to use the unbiased learning approach of CCNs to optimize prognostication and in addition to the image data only provided basic additional information of coronary segmentation and lesion localization.

The results demonstrate the feasibility of the approach. Prognostic value of the CNN algorithm alone was comparable with eoCAD and Morise score, but improved prediction significantly in combination with the others. It seems the algorithm can detect relevant prognostic information not used by standard CCTA assessment, but obviously it cannot use all information available.

Without providing coronary segmentation and lesion localization the algorithm did not improve at all, thus still requiring preprocessing of the data. The integration of fully automated lesion detection would be the logical next step of improvement. To account for the length of follow-up, it would also be relevant to use a time-to-event model in further studies.

## Limitations

The results of the present study were not externally validated on a separate cohort. The majority of our patients were males from an urban area of mainly caucasian people. Throughout the image acquisition period of 160 months four different CT scan generations were used and improvement of image quality may have affected the results. Due to the limited number of primary endpoints, we could not set aside a testing sample to train the algorithm.

## Conclusion

We developed a novel CNN model based on CCTA images to assess risk prediction for MACE and found an improved AUC when combining it with conventional CT and clinical parameters. Our results highlight the value of CNN tools in assessing CCTA images and hold great potential for further improvement in prognostication of patients with suspected CAD. In the future, we would like to identify which specific variables the CNN model had used to predict MACE. It would be also interesting to abdicate of plaque annotations or develop an automatic detection and characterization of coronary plaques and stenosis for further risk analysis tools.

## Abbreviations

ACS: Acute coronary syndrome
AUC: Area under the curve
CAC: Coronary artery calcium
CAD: Coronary artery disease
CAG: X-ray coronary angiography
CCTA: Coronary computed tomography angiography
CNN: Convolutional neural networks
CT: Computed tomography
DL: Deep learning
FRS: Framingham risk score
eoCAD: Extent of coronary artery disease
LAP: Low-attenuation plaque
MACE: Major adverse cardiac events
ML: Machine learning
SCPR: Stretched curved planar reconstructions
